# Overview of Osteoporosis and Rotator Cuff Disease

**DOI:** 10.3390/biomedicines14081789

**Published:** 2026-08-08

**Authors:** Emily T. Barr, Mina Entessari, Sarah E. Watson, Alan W. Reynolds, John P. Scanaliato

**Affiliations:** 1 Department of Orthopaedic Surgery and Rehabilitation, Atrium Health Wake Forest Baptist Medical Center, Medical Center Boulevard, Winston-Salem, NC 27157, USA; emily.barr@wfusm.edu (E.T.B.); sarah.watson@wfusm.edu (S.E.W.); alan.reynolds@wfusm.edu (A.W.R.); 2 Department of Orthopaedic Surgery, William Beaumont Army Medical Center, 18511 Highlander Medics St., Fort Bliss, TX 79906, USA; jscans@gmail.com; 3 Department of Orthopaedic Surgery and Rehabilitation, Texas Tech University Health Sciences Center El Paso, 130 Rick Francis St., El Paso, TX 79905, USA

**Keywords:** rotator cuff disease, osteoporosis, bone health

## Abstract

**Background**: Rotator cuff disease and osteoporosis are frequently overlapping pathologies in the older patient population. Increasing evidence suggests that impaired bone health negatively impacts rotator cuff tear development and healing after repair. **Methods**: An online search of PubMed and MEDLINE was performed from inception to April 2026, using the keywords “osteoporosis” and “rotator cuff”. Thirty-two studies met all criteria. These studies summarize the current literature on the relationship between osteoporosis and rotator cuff disease, with an emphasis on epidemiology, evaluation, treatment implications, and future directions. **Results**: Osteoporosis is associated with rotator cuff tear development, poorer local bone quality, and impaired tendon-to-bone healing following repair. Chronic rotator cuff pathology may also contribute to localized osteopenia of the greater tuberosity, suggesting a bidirectional relationship between bone loss and rotator cuff degeneration. Despite this overlap, osteoporosis remains under-recognized and under-treated in patients with rotator cuff disease. Vitamin D status, bone health assessment, and emerging bone-active therapies have demonstrated improved outcomes. **Conclusions**: Osteoporosis is an important comorbidity to consider in patients with rotator cuff disease. Improved recognition and optimization of bone health may enhance both skeletal and shoulder-specific outcomes. Further prospective studies are needed to better define optimal management strategies.

## 1. Introduction

Rotator cuff disease encompasses a spectrum of acute and chronic pathology involving the subscapularis, supraspinatus, infraspinatus, and teres minor, as well as their tendinous insertions on the proximal humerus. Shoulder pain is one of the most common musculoskeletal complaints in older adults, and rotator cuff disease accounts for a substantial portion of this burden. In a population-based ultrasonographic study of 1366 shoulders, full-thickness rotator cuff tears were identified in 20.7% of the general population, with prevalence increasing markedly with age [1]. While rotator cuff pathology in younger individuals may result from trauma, overuse, or other extrinsic mechanical factors, in older adults, it is more commonly associated with intrinsic tendon degeneration and age-related tissue changes [1]. Natural history studies have shown that degenerative tears frequently enlarge over time and that tear progression is associated with the development of new pain and symptoms; in a prospective longitudinal cohort, tear enlargement was observed in 49% of shoulders at a median of 2.8 years, with new pain developing in nearly half of subjects [2]. Consistent with these findings, incidental rotator cuff derangement on magnetic resonance imaging (MRI) is highly prevalent and has been reported to be nearly universal after age 40 years, present in 98.7% of shoulders in a recent population-based MRI study [3].

As with rotator cuff disease, osteoporosis is a common age-related condition that becomes increasingly significant with advancing age. Osteoporosis is a systemic skeletal disease characterized by low bone mass and microarchitectural deterioration, typically defined by a bone mineral density (BMD) at least 2.5 standard deviations below that of a healthy young adult reference population [4,5]. In the general population, the prevalence of osteoporosis has been estimated at approximately 18% to 20%, while osteopenia affects roughly 40% to 43% of individuals [4,5]. Importantly, the prevalence of osteoporosis rises sharply after the age of 50, resulting in a disproportionate burden among older adults—coinciding with the demographic in which degenerative rotator cuff pathology is most frequently observed [4,5].

Beyond sharing a common demographic profile, osteoporosis appears to be independently associated with both the development of rotator cuff tears and worse outcomes following attempted repair. In a large longitudinal cohort study, patients with osteoporosis had a 1.79-fold higher risk of developing a rotator cuff tear than those without osteoporosis, an association independent of age and sex [6]. Conversely, among patients with rotator cuff tears, the prevalence of osteoporosis has been reported to be as high as 61%, markedly exceeding that of the general population, while only 12.9% of affected patients were receiving osteoporosis treatment in one recent series [7]. Collectively, these findings suggest that osteoporosis is not merely a coincident feature of aging but a clinically relevant factor that may contribute to both the onset of rotator cuff pathology and the risk of complications after surgical repair.

Though there is a clear epidemiologic relationship between osteoporosis and rotator cuff disease, and osteoporosis has been identified as a risk factor for rotator cuff tear development, the current evidence remains fragmented across different studies. There remains a lack of comprehensive reviews synthesizing the current literature on the relationship and clinical implications of osteoporosis and rotator cuff disease. Given the aging population and continued growth of both these conditions, a consolidated review can better inform clinicians while caring for these patients. The purpose of this narrative review is to provide an overview of the evaluation, treatment, management, and outcomes of osteoporosis and rotator cuff disease.

## 2. Methods

A literature search was performed in April 2026 of the existing literature regarding osteoporosis and rotator cuff disease. The search utilized the electronic databases PubMed and MEDLINE from inception to April 2026. Keywords used in the search were “osteoporosis” and “rotator cuff”. Inclusion criteria were English-language studies focusing on rotator cuff disease or osteoporosis alone, or the relationship between rotator cuff disease and osteoporosis. Exclusion criteria included case reports and studies that did not evaluate rotator cuff disease or osteoporosis. A total of 185 articles were identified in the initial search; 32 articles satisfied all criteria and were utilized in this narrative review. The main outcome variables of interest were epidemiology, evaluation, treatment, management, outcomes, and future directions.

## 3. Epidemiology

Age-stratified epidemiologic studies further clarify the burden of rotator cuff tears in older adults. In an ultrasonographic survey of asymptomatic shoulders, Tempelhof and colleagues reported full-thickness tears in 13% of patients in their sixth decade, 20% in their seventh, 31% in their eighth, and 51% in those older than 80 years [8]. Yamamoto and colleagues similarly identified full-thickness tears in 12.8% of shoulders in the fifth decade, 25.6% in the sixth, 45.8% in the seventh, and 50.0% in the eighth decade, confirming a marked age-dependent increase in tear prevalence [1]. Taken together, these data suggest that approximately 10% to 20% of adults over age 50 already harbor a rotator cuff tear, with prevalence rising substantially with each successive decade and the odds of harboring a tear increasing by roughly 2.7-fold per decade of life [1,8].

Within this age-related framework, osteoporosis appears to represent more than a parallel manifestation of aging. Hong and colleagues demonstrated, in a population-based cohort of more than 21,000 patients followed for 7 years, that osteoporotic patients had a significantly greater cumulative incidence of rotator cuff tears (adjusted hazard ratio, 1.79; 95% confidence interval (CI), 1.55–2.05), with no significant interaction by age or sex, suggesting that metabolic bone disease may independently contribute to tear development [6]. More recently, Liu and colleagues supported this relationship in a UK Biobank analysis of more than 450,000 participants, demonstrating that osteoporosis was associated with both higher cross-sectional prevalence (adjusted odds ratio, 1.38; 95% CI, 1.25–1.52) and higher longitudinal risk of rotator cuff tears (adjusted hazard ratio, 1.56; 95% CI, 1.29–1.87), while colocalization analyses identified shared genetic loci—including the *PKDCC* rs12996954 variant—implicating common degenerative and molecular pathways linking bone loss and cuff pathology [9].

This relationship may also be self-reinforcing at the local level. Cadet and colleagues demonstrated significantly increased greater tuberosity osteopenia in chronic, retracted tears as compared with acute, minimally retracted tears, supporting the concept of a vicious cycle in which impaired systemic bone quality predisposes to tearing, while chronic rotator cuff insufficiency further accelerates local bone loss at the tuberosity [10]. The high prevalence of unrecognized and untreated osteoporosis in patients with rotator cuff tears—reported in as few as 12.9% of cases despite a prevalence approaching 60%—underscores a substantial care gap and a missed opportunity for both skeletal and shoulder optimization in this population [7].

## 4. Evaluation

Evaluation of the patient with suspected rotator cuff disease begins with a thorough history and physical examination. Key historical features include the chronicity and mechanism of symptoms, dominant arm involvement, occupational and recreational demands, prior shoulder injury or surgery, and response to nonoperative measures. A history of antecedent trauma, sudden loss of strength, or progressive nocturnal pain raises concern for a clinically significant tear, while an insidious onset in an older patient is more consistent with degenerative pathology [1]. Given the high prevalence of incidental rotator cuff abnormalities on advanced imaging—with cuff tendon abnormalities now reported in nearly 99% of shoulders in adults aged 41 to 76 years—imaging findings must always be interpreted within the clinical context, as the majority of MRI-detected abnormalities may not be the source of symptoms [3]. Physical examination should systematically assess the patient’s range of motion, strength testing of each rotator cuff muscle, and provocative maneuvers, as well as inspection for atrophy of the supraspinatus and infraspinatus fossae, which suggests chronicity. Differentiating rotator cuff pathology from adhesive capsulitis, glenohumeral arthritis, cervical radiculopathy, and other shoulder girdle pathology is essential before proceeding with advanced imaging or invasive treatment.

Decision-making between operative and nonoperative management benefits from an appraisal of established predictors of nonoperative success, as outlined in the Multicenter Orthopaedic Outcomes Network (MOON) Shoulder Group studies and other prospective cohorts [11]. Tear chronicity, baseline patient-reported function, comorbidity burden, and patient activity demands all influence the likelihood of durable nonoperative response, and shared decision-making is appropriate in patients with degenerative tears amenable to a structured rehabilitation trial. In contrast, primary tendon repair has demonstrated superior long-term functional and structural outcomes for small to medium-sized tears in appropriately selected patients, with mean Constant scores 9.6 points higher and American Shoulder and Elbow Surgeons scores 15.7 points higher than physiotherapy at 10 years in a randomized comparison [12].

Particular attention should be paid to age-related comorbidities that interact with both rotator cuff disease and bone quality. Sarcopenia and frailty—progressive losses of skeletal muscle mass, strength, and physiologic reserve—commonly co-occur with osteoporosis and rotator cuff degeneration, contributing to diminished strength, impaired healing potential, and increased perioperative risk [13]. Frailty assessment, whether by validated screening instruments or by simple measures of grip strength, gait speed, and unintentional weight loss, can help stratify surgical candidacy and guide preoperative optimization [13].

Medication reconciliation should likewise be integrated into the standard rotator cuff workup, including review of chronic glucocorticoid use, antiresorptive therapy, and emerging classes such as glucagon-like peptide-1 (GLP-1) receptor agonists. Recent data raise concern that GLP-1 agonist–associated weight loss may adversely affect bone mineral density, although current evidence does not suggest a deleterious effect on rotator cuff repair outcomes specifically [14]. Glycemic optimization should also be confirmed in patients with diabetes, as elevated perioperative hemoglobin A1c levels above approximately 8.0% have been associated with a roughly two-fold increase in postoperative infection following arthroscopic rotator cuff repair [15].

When osteoporosis is suspected or known, evaluation should be expanded beyond the shoulder. On standard anteroposterior radiographs, the deltoid tuberosity index (DTI)—the ratio of the outer cortical width of the proximal humerus to the cortical width at the surgical neck—provides a simple, validated surrogate for proximal humeral bone quality and correlates with dual energy X-ray absorptiometry (DEXA)-derived BMD; lower DTI values have been associated with poorer bone purchase and higher complication rates following shoulder surgery [16]. Because the proximal humerus is non-weight-bearing, hip and spine DEXA may underestimate local BMD at the greater tuberosity, particularly in obese patients, and a normal central DEXA does not exclude clinically significant osteopenia at the cuff footprint [17].

Particular attention should be paid to patients with risk factors for impaired bone health—postmenopausal status, low body mass index (BMI), prior fragility fracture, chronic glucocorticoid use, family history of osteoporosis, malabsorptive states, hypogonadism, or other endocrinopathies—as these patients may require additional subspecialist evaluation and treatment [17]. Furthermore, serum 25-hydroxyvitamin D should be considered in patients undergoing evaluation for rotator cuff repair, as vitamin D deficiency (defined as 25-hydroxyvitamin D < 20 ng/mL) has been reported in 36% to 47% of the general U.S. and European populations and is even more prevalent among patients with fragility fractures, with potential implications for tendon healing and muscle quality [17].

A practical algorithm for the shoulder surgeon is therefore to obtain plain radiographs (with calculation of the DTI when bone quality appears compromised), consider serum 25-hydroxyvitamin D or perioperative supplementation in all patients being considered for rotator cuff repair, and refer for DEXA and endocrinology consultation when osteoporosis is suspected based on clinical risk factors, prior fragility fracture, or suggestive imaging findings [16,17]. Despite the high prevalence of osteoporosis in this population—reaching 61% among patients with documented rotator cuff tears in one recent series—only 12.9% of affected patients were receiving osteoporosis treatment, highlighting a substantial care gap and a clear opportunity for orthopaedic surgeons to facilitate appropriate medical management [7].

The role of pharmacologic osteoporosis therapy, specifically as a strategy to reduce rotator cuff repair complications, remains less well defined. Animal studies of antiresorptive agents have demonstrated reductions in osteoclast density at the tendon-bone interface and improvements in the biological healing milieu in models of rotator cuff repair, providing biologic plausibility for a benefit [18]. Clinical data, however, have not consistently demonstrated reductions in revision rotator cuff surgery rates among patients receiving bisphosphonate therapy at the time of repair [19]. Based on the current evidence, bone health should be optimized in osteoporotic patients undergoing rotator cuff repair through a multidisciplinary approach—in keeping with the principles of fracture liaison and “Own the Bone” style care pathways—for overall skeletal health and fracture prevention [20,21]. The decision to initiate or continue antiresorptive therapy specifically to mitigate surgical complications should be individualized; the value of such therapy is not binary but depends on the timing of surgery and the available window for optimization.

## 5. Treatment and Management

The management of patients with concomitant osteoporosis and rotator cuff disease is multifactorial, with a focus on both optimizing bone quality and maximizing shoulder function. It bears mentioning that while osteoporosis treatment may not have an immediate effect on patient outcomes following either operative and nonoperative management of rotator cuff disease, it is focused on minimizing the risk of adverse skeletal events secondary to reduced bone mineral density. As previously mentioned, despite the high incidence (and substantial overlap) of both rotator cuff disease and osteoporosis, the screening for and treatment of osteoporosis in patients seen for rotator cuff disease is woefully underperformed [7,22].

### 5.1. Management of Osteoporosis

While a detailed overview of the medical management of osteoporosis is beyond the scope of this article, we feel a cursory overview will be helpful information for those who routinely treat patients with rotator cuff disease. Treatment is best administered through a multidisciplinary team approach and should focus on ameliorating modifiable risk factors, supplementation with calcium and vitamin D, and, when indicated, initiation of osteoporosis-specific pharmacotherapy. Some hospitals and healthcare systems have begun establishing and using a fracture liaison service, which can help patients coordinate care with providers across specialties to streamline evaluation and treatment [21].

Numerous risk factors for the development of osteoporosis have been identified. While some are beyond the control of the patient, many are modifiable, and addressing these risk factors can have a profound effect on a patient’s risk of experiencing an osteoporosis-related fracture. A dietary consultation can identify any nutritional deficiencies and ensure that patients are consuming enough calories with the right balance of nutrients, minerals, and vitamins [23]. Patients should be counseled to cease the utilization of tobacco and/or nicotine products and abstain from alcohol consumption [24,25]. Physical activity, especially weight-bearing cardiovascular and resistance training, should be encouraged, as these modalities preserve lean body mass and BMD and make patients more resistant to falls and/or loss of balance. It is important to note, however, that excessive and/or aggressive weight loss should be avoided. Calcium and vitamin D therapy should be initiated in all patients, and the current recommendation is between 1200 and 1500 mg/day of calcium and 800–1000 IU of vitamin D for patients over the age of 50. The addition of these two over-the-counter supplements is widely supported in the literature, with the addition of calcium demonstrating a 34% reduction in the rate of osteoporosis-related fractures and high-dose vitamin D demonstrating a 24% reduction in the rate of hip fractures and a 30% reduction in the rate of all other non-vertebral fragility fractures. With respect to rotator cuff disease, optimization of vitamin D levels is crucial, as preoperative levels < 30 ng/mL are predictive of increased fatty degeneration and a higher risk of postoperative adverse events, and preoperative supplementation with vitamin D can both reduce revision rates and overall health care burden in patients undergoing arthroscopic rotator cuff repair [26].

Regarding pharmacologic treatment, current guidelines recommend the initiation of medications in any patient who has sustained a fragility fracture or a patient who has a T-score < −2.5 at the femoral neck or spine or a T-score between −1.0 and −2.5 at the femoral neck or spine with a 10-year risk of hip fracture ≥ 3% or a 10-year risk of major osteoporosis-related fracture (as per the Fracture Risk Assessment Tool FRAX score) ≥ 20%. The first-line pharmacologic agents of choice for most patients are bisphosphonates, antiresorptive medications that inhibit osteoclastic bone resorption, although some patients may undergo treatment with anabolic agents, e.g., Teriparatide, Abaloparatide, Romosozumab [27]. Although the focus of pharmacologic treatment is the prevention of future fractures, certain agents may confer benefit for rotator cuff disease. A recent triple-blind randomized controlled trial in 43 patients aged 50–80 years with degenerative rotator cuff tears demonstrated that Teriparatide significantly improved functional recovery and tendon-to-bone healing following rotator cuff repair [28]. This level 2 study provides high-quality evidence to support the use of anabolic bone agents to improve outcomes after degenerative rotator cuff tear repair in patients over the age of 50 [28]. Denosumab has been found to be protective against retear in osteoporotic women over the age of 60 and can lower retear rates to levels comparable to non-osteoporotic controls [29].

### 5.2. Management of Rotator Cuff Disease

The treatment of rotator cuff disease in patients with osteoporosis is similar to that in patients with normal BMD. Acute tears associated with a discrete loss of or alteration in function are often treated with early surgical intervention in the form of rotator cuff repair. Chronic rotator cuff pathology is often initially managed through conservative means, with a focus on activity modification, anti-inflammatory medications, and structured and progressive physical therapy.

### 5.3. Nonoperative Management

Nonoperative management of rotator cuff disease is focused on pain reduction, strength maximization, and motion optimization. Successful nonoperative management hinges on restoring balanced force couples across the glenohumeral joint, optimizing scaption, and recruiting compensatory muscles [30]. There has been extensive research on predicting which patients will succeed with nonoperative management of rotator cuff disease [11]. Overall, despite a high comorbid burden, the presence of osteoporosis does not appear to be a risk factor for the failure of nonoperative management.

### 5.4. Operative Management

Acute rotator cuff tears or chronic rotator cuff tears with symptoms recalcitrant to appropriate nonoperative management are often treated surgically with rotator cuff repair. While the effect of osteoporosis on the outcomes of nonoperative treatment of rotator cuff disease appears to be minimal, at worst, the same cannot be said with respect to operative management. Overall, osteoporosis has been identified as an independent risk factor for healing failure following rotator cuff repair. Osteoporosis, regardless of treatment status, has been identified as a risk factor for revision following rotator cuff repair [31]. Localized osteopenia of the greater tuberosity, secondary to increased osteoclastic activity, can affect anchor pullout strength and has been associated with altered biology at the tendon enthesis [32]. In patients with a history of fragility fracture or chronic steroid use, BMI < 20, and females > 60 and males > 70, a preoperative computed tomography (CT) scan to determine greater tuberosity BMD can be considered, as <120 Hounsfield units (HU) has been associated with a high risk of anchor failure [7].

### 5.5. Preoperative Optimization

Limited evidence exists on the benefit of preoperative antiresorptive therapy for patients undergoing rotator cuff repair. While rat models suggest potential clinical benefit from a variety of osteoporosis medications, human database studies call these findings into question. The evidence for vitamin D supplementation, however, is much more robust. Vitamin D deficiency is associated with higher rates of postoperative complications following rotator cuff repair, including retear rates, revision rates, and pain [33]. A proposed algorithm for management of osteoporosis and rotator cuff disease by the authors is demonstrated in Table 1.

### 5.6. Surgical Technique

As a result of the increased revision rate and retear rate following rotator cuff repair in osteoporotic patients, numerous strategies have been proposed and reported in order to optimize the repair construct. BMD has been demonstrated to be an independent risk factor for successful tendon healing. The rotator cuff healing index (RoHI) predicts a lower likelihood of successful tendon healing in patients with BMD ≤ −2.5 [34]. Metallic or threaded anchors may confer additional resistance to pullout compared to other anchor designs. Anchors should be inserted perpendicular to the tuberosity surface. Anchor holes can be punched partially, and the anchor itself can be used in a self-tapping technique. Surgeons should consider using additional anchors to distribute stress across multiple fixation points and may even consider an anchorless transosseous technique [35]. Decortication of the greater tuberosity footprint should be kept to a minimum or outright avoided. Anchor holes can be augmented with bone graft and/or cement [36].

**Table 1:** Recommended algorithm for treatment of osteoporosis and rotator cuff disease.

Recommended Algorithm
**Assessment:** Screen for osteoporosis in all rotator cuff tear patients ≥ 65 years (women) or ≥70 years (men), or younger patients with risk factors Obtain DEXA of hip and lumbar spine when indicated Consider assessment of vitamin D status and nutritional markers
**Treatment Planning:** For nonoperative candidates with osteoporosis Initiate exercise program and rotator cuff focused physical therapy Consider corticosteroid injection for symptomatic relief If available, referral to a fracture liaison service for osteoporosis medical management 2. For surgical candidate with osteoporosis Consider preoperative optimization with an anabolic agent when appropriate Consider modified surgical techniques Plan for potential intraoperative anchor failure Discuss postoperative bisphosphonate therapy or referral
**Postoperative Management:** Can consider zoledronic acid 5 mg IV on postoperative day 1 and at 1 year Optimization of vitamin D and calcium intake/supplementation Standard rehabilitation protocol with emphasis on fall avoidance to minimize fracture risk Standard interval follow-ups
**Long-term Follow-up:** Multidisciplinary management of osteoporosis Monitor BMD with DEXA Fall prevention education and strategies Ongoing shoulder rehabilitation and maintenance exercises

Key: DEXA, dual-energy X-ray absorptiometry scan; IV, intravenous; BMD, bone mineral density.

## 6. Outcomes

Osteoporosis has been demonstrated to be a poor prognostic factor following rotator cuff repair. In a database study, Scott et al. found that osteoporotic patients (mean age 66.4 and 87.1% female) had significantly higher rates of pathological humerus fracture and conversion to reverse total shoulder arthroplasty two years after repair [37]. These patients also had more postoperative complications, including anemia, shoulder stiffness, and greater healthcare utilization, within 90 days after rotator cuff repair [37]. Similarly, Chung et al. reported higher rotator cuff repair failure rates at lower BMD: 9% for T-scores > −1.0, 30.2% for T-scores between −2.5 and −1.0, and 41.7% for T-scores < −2.5 [31]. Not all findings agree; Sierra et al. reported no difference in two-year postoperative patient-reported outcomes following rotator cuff repair between patients with a mean age of 60 and 53.5% male, with or without osteoporosis [38].

Pharmacologic agents for osteoporosis have shown mixed effects on rotator cuff tendon healing. In osteoporotic patients, zoledronic acid administration after rotator cuff repair was associated with significantly lower retear rates at six months postoperatively compared to patients who did not undergo therapy, 13.3% vs. 30.3%, respectively [39]. Other studies show no benefit. In a prospective observational study of females over the age of 60 receiving denosumab therapy after repair, Kim et al. found no significant difference in functional scores or retear rate at six months between osteoporotic and non-osteoporotic patients [29].

It is important to recognize the heterogeneity in the literature regarding the relationship between osteoporosis and rotator cuff disease. Several gaps remain in the literature regarding the exact pathophysiology and management recommendations due to heterogeneity across studies in patient characteristics, definition of osteoporosis, surgical techniques, outcome assessment, and follow-up. As such, given the lack of prospective studies and randomized controlled clinical trials in the literature, direct conclusions cannot be drawn.

## 7. Future Directions

Future work should clarify the biologic mechanisms linking osteoporosis and rotator cuff disease, which appear to share degenerative, molecular, and musculoskeletal pathways [9]. Better mechanistic understanding could sharpen risk stratification, helping clinicians identify patients at higher risk for tear development, tear progression, or impaired healing after repair. Basic science studies focused on the molecular pathways linking bone loss and tendon degeneration would provide a better understanding of the pathophysiology between osteoporosis and rotator cuff disease.

The role of vitamin D remains poorly defined. Deficiency has been linked to poorer muscle quality, increased fatty degeneration, and more adverse events following shoulder surgery. The current literature remains heterogeneous regarding threshold values, timing of supplementation, and whether correcting deficiency improves structural healing or only reduces complications [17,26,33]. Prospective studies should define standardized perioperative screening, clinically meaningful target levels, and whether preoperative supplementation reliably improves tendon healing, lowers retear rates, and enhances patient-reported outcomes after rotator cuff repair [26,33]. In particular, a randomized controlled study comparing tendon healing rates in osteoporotic patients who undergo rotator cuff repair, with and without various doses of vitamin D, would provide more evidence-based recommendations on vitamin D supplementation for patients undergoing rotator cuff repair.

Anabolic and other bone-active agents are another priority for study in rotator cuff repair. Preclinically, anti-osteoporotic therapies improve tendon-to-bone healing through effects on osteoclast activity, local bone preservation, and the biologic environment of the enthesis [18,32]. Early clinical data are also encouraging: teriparatide has demonstrated improved functional recovery and tendon-to-bone healing in a randomized trial, and denosumab brought retear rates in older osteoporotic women close to those of non-osteoporotic controls [28,29]. Optimizing bone metabolism may therefore aid rotator cuff healing in selected patients as well as prevent fracture, but key questions remain: which patients benefit most, which agents are most effective, the optimal timing and duration of therapy, and whether benefits vary with tear size, chronicity, revision status, or local bone loss at the greater tuberosity. Future work should prioritize prospective multicenter trials that integrate bone phenotype, tear characteristics, and medication timing to define the role of anabolic and other bone-active therapy in routine perioperative rotator cuff care [18,28,29,32].

## 8. Conclusions

Osteoporosis has been demonstrated to be a risk factor for rotator cuff tears and a poor prognostic factor after repair. Bone health optimization should be considered throughout the evaluation and management of rotator cuff pathology, particularly in older patients. This calls for a multidisciplinary approach spanning appropriate screening, medical referral, nutritional optimization, and surgical planning tailored to bone quality. Prospective studies are needed to define the roles of vitamin D optimization, genetic and molecular risk stratification, and anabolic or other bone-active therapies in improving rotator cuff outcomes.

## Data Availability

No new data were created or analyzed in this study.

## Abbreviations

The following abbreviations are used in this manuscript:

MRI	Magnetic resonance imaging
BMD	Bone mineral density
CI	Confidence interval
MOON	Multicenter Orthopaedic Outcomes Network
GLP-1	Glucagon-like peptide-1
DTI	Deltoid tuberosity index
DEXA	Dual energy X-ray absorptiometry
BMI	Body mass index
FRAX	Fracture Risk Assessment Tool
RoHI	Rotator cuff healing index
